# How near-peer supervisors experience their own development in the supervisor role when shifting from written to oral interactive feedback: a qualitative interview study

**DOI:** 10.1186/s12909-025-07798-0

**Published:** 2025-08-16

**Authors:** Anna Victoria Flankegård, Julie Solberg Knutsen, Eivind Alexander Valestrand, Knut Eirik Ringheim Eliassen

**Affiliations:** 1 Section for General Practice, Department of Global Public Health and Primary Care, University of Bergen, Bergen, Norway; 2 Akershus University Hospital, Lørenskog, Norway

**Keywords:** Peer teaching, Student supervisors, Feedback competency, Professional identity formation, Medical students, Interactive feedback.

## Abstract

**Background:**

Oral interactive feedback has been shown to help students learn more compared to written feedback. However, less is known of what student supervisors gain from engaging in feedback with peers or near peers. This study explored near-peer supervisors’ experiences of development in their role as supervisors when shifting from one-way written feedback to oral interactive feedback.

**Methods:**

We conducted a qualitative interview study with 10 medical student supervisors from an introductory course in person-centred medicine. In focus groups, they reflected upon how they experienced the difference between providing written and oral interactive feedback, and what impact it had on them. Analysis was conducted with systematic text condensation, a method for thematic cross-case analysis.

**Results:**

The supervisors reported that oral interactive feedback contributed to their development in various aspects of their supervisor role. Through feedback dialogues with students, they gained confidence and experienced both professional and personal growth. They also learned to adapt the feedback methods to different purposes. Additionally, they found their supervisor experiences relevant to meeting patients as physicians, describing improvement in communication skills, leadership and the ability to establish professional relationships, as well as acquiring new insights into their future role as physicians.

**Conclusions:**

Institutions should recognise a potential reciprocal gain for both parts in a feedback process and consider facilitating oral interactive feedback more frequently, not least in education encouraging students to reflect upon own experiences and emotions. Providing oral interactive feedback can be a powerful learning opportunity. Student supervisors should be encouraged to explore the close connection between their role as supervisors and their future role as physicians.

**Supplementary Information:**

The online version contains supplementary material available at 10.1186/s12909-025-07798-0.

## Background

Students consistently seek feedback, yet often find it lacking in both quality and frequency [1, 2]. In Norway, medical students are clearly the least satisfied with the feedback they receive [2]. While employing students as supervisors for their peers has been shown to increase understandable, meaningful and relevant feedback [1, 3], there still exists less literature about learning outcomes for the student supervisors.

Traditionally, feedback has been described as something you give or receive, but more recent literature describe a shift in the understanding to feedback being seen as a learning-centred process where learners also must take an active role [4, 5]. To create productive feedback, there should be an educational alliance between the two parts of the feedback process [5]. Peers are often perceived as less intimidating than experts and may be better positioned to create a motivating, safe learning environment [6, 7]. The same should apply for near peers, meaning peers with one or more years educational distance. This safe learning environment positively influences how well students engage with the feedback [8, 9]. Also, it is easier to discuss challenges with peers, who can better relate to their experiences than faculty members [6, 7]. The difference can be attributed to greater social and cognitive congruence among peers who share similar social standings, knowledge bases, and thought processing [3, 6, 10]. Consequently, peers may offer feedback on a more suitable cognitive level and better express understanding of the learner’s needs and concerns [3].

Using peers also gives more frequent opportunities for dialogical feedback. Feedback in dialogue is shown to enhance feedback learning outcomes, since it increases the likelihood for realisation of other feedback recommendations, such as selectivity, specificity and optimal timing of the feedback [1]. Dialogue also makes it easier to adapt language to the other person and to the context, hence lowering risks for misunderstandings [1, 5]. Additionally, activating peers as supervisors can help fulfilling the need for continuity in the student-supervisor relationship and for feedback to be an ongoing, iterative dialogue, when large student numbers make one-to-one student-teacher conversations not feasible [1].

Dialogical feedback can be provided in either written or oral format, each with distinct advantages. Written feedback often offers clarity and details, while oral feedback allows for real-time negotiation and reflection [1]. Facilitating reflection is a vital part of the task as a supervisor, because it guides the other in the right direction of self-assessment [5]. The feedback provider’s own self-reflection is also important, because it improves their feedback skills, leading to gains for both themselves and their feedback partner [8].

Peer teaching and peer feedback have been shown to be beneficial for both the provider and the receiver [1, 11–13]. A study from Scotland in 2014 reported that producing feedback is just as valuable for learning as receiving feedback, because students constructing feedback gain a deeper understanding of the subject and acquire new skills [12]. Students acting as teachers do not only ‘learn twice’, but also learn in a different way, that adds to their knowledge in a meaningful way [6, 7].

This study explored the experiences of near-peer supervisors when shifting from written to oral interactive feedback. We sought to answer: *How did the supervisors experience the difference between written and oral interactive feedback*,* and what impact did it have on their own development in the supervisor role?*

## Methods

### Study design

This is a qualitative study employing a socio-constructive paradigm with a pragmatic approach and convenience sampling. Focus group discussions were chosen for data collection. We asked student supervisors about their experiences and development in their own supervisor role when shifting from one-way written feedback to oral interactive feedback. By oral interactive feedback, we mean feedback provided in an oral, two-way dialogue, where both sides have active roles, and the supervisor stimulates the learner to reflect.

### Context

During the first year of medical school at the University of Bergen, Norway, students attend a mandatory course called *Patient contact* – PASKON. The aim of PASKON is to prepare and encourage students for person-centred medicine and help them develop an understanding of how disease and sickness can affect a patient’s life and how physicians can make a difference for people with various health complaints and conditions. The course gives an introduction to communication and consultation techniques, such as the use of open-ended questions at the beginning of patient interactions, active listening, and actively seeking answers to how the health problem looks from the patient’s perspective [14, 15].

Groups of 5–6 students visit a seriously ill patient, unaccompanied, typically in the patient’s home, to gain insight into how this person is affected by his/her sickness and how it can feel to be a patient. Thereafter, the group convene in class together with the patient and share the main storylines in an interactive plenary session. Although meeting only one patient on their own, students have close encounters with 20 patients in the plenary sessions throughout the course. Furthermore, the students are asked to write three individual reflective essays based on their patient meetings. The first two are written shortly after the patient meeting and encourage the students to reflect on *‘How does the patient’s health problems affect his/her life?’* and *‘How was it to be me in the role of a medical student in my first professional patient encounter?’* At the end of the course, they write a larger third reflective essay, where they can choose from different themes [16].

Every group of first year students are connected to a senior student acting as their supervisor. One student supervisor is responsible for two groups. The role of the student supervisors is to support their groups before and after their patient meeting, and to provide individual feedback on the reflective essays. The supervisors were trained for the job through two afternoon workshops. The first workshop included role modelling of group formation and dynamics. Building relationships and emphasising on exploring expectations, both for students and supervisors, were also topics for this workshop. The second workshop was dedicated to written and oral interactive feedback. In an interactive session they reflected together with each other and the workshop leaders on the concept of feedback, as well as the strengths and weaknesses of both written and oral interactive feedback. Furthermore, they learned what characterises reflection, received tips on how to provide effective feedback according to recent research, and practiced oral interactive feedback with each other.

Originally, the supervisors were trained in how to provide feedback as written, one-way feedback, but also encouraged to set the stage for two-way communication by writing reflective questions. For the academic year 2023/2024, the feedback approach for PASKON was changed, instructing the student supervisors to keep written feedback on the first essay, and to facilitate oral interactive feedback for each student around the second essay. For the third reflective essay the supervisors could choose between written and oral interactive feedback.

### Data collection

In total, 10 out of 15 student supervisors were interviewed in three groups in the period January to April 2024. Inclusion criterion was having the role as a student supervisor in the PASKON course in 2023/2024, regardless of earlier experiences (informant details in Table 1). AVF administrated and facilitated all interviews, while JSK and KEE joined as co-moderators. The moderators used a semi-structured interview guide (Supplementary material 1), and the interviews lasted about 90 min each. The interviews were audio-recorded, and then transcribed verbatim and anonymised by Semantix Translations Norway [17].

**Table 1:** Distribution of informants

	Informants^1^
Interview group 1	4 females + 1 male
Interview group 2	1 female + 2 males
Interview group 3^2^	2 females

^1^To preserve anonymity, citations in the results are signed only with supervisor number, due to gender distribution and the varying numbers of informants in the interview groups

^2^ Planned as a focus group. However, only two informants showed at the set time

The reason for choosing focus groups as data collection method was that we explored a phenomenon that the informants have a shared lived experience of, though being a part of their individual developments as student supervisors. The topics discussed in the focus groups were not considered sensitive or very personal. Furthermore, we had reason to believe the informants felt safe in each other’s presence to speak freely, both as the informants were peers or near peers to each other and as the training also had a relational effect within the student supervisor group. Since the phenomenon we explored was from a quite small change in practice, we expected that interviewing informants in focus groups rather than individually, could have a beneficial effect, as informants could build upon each other’s statements.

### Data analysis

Data were analysed using systematic text condensation (STC), a method used for thematic cross-case analysis of qualitative data [18]. Analysis was carried out by AVF in cooperation with the other authors. STC consists of the following steps: 1) get a general impression by reading the data and suggest preliminary themes, 2) identify meaning units and sort them into code groups, 3) summarise the contents of the meaning units of each code group to form condensates and 4) synthesise the condensates to make an analytical text, validate the results and make descriptions and concepts [18]. The authors used an “all in-reaction” writing strategy [19].

### Reflexivity

As a group we present a variety of competencies, being a medical student (AVF), educational researchers (JSK, EAV, KEE), and medical doctors (JSK, EAV, KEE). Together, we share research interests in feedback, professional identity formation, supervision, and faculty development.

All authors have a strong connection to the PASKON course, with experience from being students (AVF, JSK), supervisors (AVF, JSK, EAV), and educators (JSK, EAV, KEE) in the course. In addition, AVF wrote an essay about being a student supervisor in PASKON during an elective course in medical education. The ideas presented in that essay became a springboard to make the changes in PASKON that we study in this project. With this closeness to the course and the changes made to it, it was important for us to be aware of our positionality, theoretical and methodological assumptions, and how we interacted with the study informants. One of the authors (EAV) was therefore more distanced from the data collection and analysis, ensuring that we had a group member positioned to question the research process and findings through the research process.

## Results

The student supervisors experienced that oral interactive feedback promoted their development in different aspects of their role as supervisors. They understood that both written and oral interactive feedback were useful in their own ways, and that it impacted the supervisor role. Some said that it was more demanding to facilitate oral interactive feedback, but that they in return learned more from it, both professionally and personally. Even though the supervisors had different focuses during their conversations, they saw several connections between supervision of students and meeting patients in their future role as physicians. They discovered that they had learned how to engage in more effective feedback, thereby enhancing their supervisor competency. In addition, the supervisors also reflected on logistical challenges and about how the focus group interviews functioned as additive reflective rooms.

### The supervisors developed throughout the course of feedback sessions

The supervisors experienced development in their role as supervisors throughout the oral interactive feedback sessions. Initially, they had felt uneasy and believed they lacked sufficient experience and knowledge. However, as they experienced arranging oral interactive feedback, they discovered that they could approach the rest of the feedback sessions with greater calmness and confidence. When discovering that everyone was worried and concerned about the same things when entering a conversation like this, the focus shifted from an internal view to a student-centred view. Increased experience reduced their discomfort, with one supervisor describing it as overcoming a threshold, becoming more comfortable in the discomfort.

*‘I was nervous before the first oral feedback. Then I provided the feedback*,* and then I looked forward to the rest of the feedback sessions. It was just a bit like that*,* a threshold.’* (Supervisor 1).

Several supervisors reported that their supervisor competency improved continuously through their experiences with oral interactive feedback. This mode of feedback facilitated tailoring feedback individually to each student, making conversations more unique compared to the written feedback. The supervisors noted the need to prepare not only by reading the reflective essays, but also by considering how to meet each single student in the feedback dialogues. They learned that oral interactive feedback served different purposes than written feedback, as it allowed for broader perspective and created opportunities for reflection rather than focusing on details and written structure. Some supervisors also explained that with increasing experience, identifying central themes became easier.

*‘(…) It was a couple of things that reoccurred and that I felt worked in the conversation and was worth spending time on. So maybe I became more and more aware of what really was important to emphasise in the conversations. Not just everything*,* but perhaps more central things.’* (Supervisor 5).

### Facilitating oral interactive feedback offered the supervisors both personal and professional outcomes

The supervisors stated that providing oral interactive feedback led to professional development by increasing awareness of the supervisor role. They felt more focused and engaged during the conversations. Some students explained it was easier to show empathy, compared to written feedback which seemed harder and colder.

*‘I found it somewhat strange to provide written feedback on reflection note 3*,* when my students shared a lot of vulnerable content in reflection note 2*,* for which I gave oral feedback. It was like closing the doors.’* (Supervisor 9).

Oral interactive feedback also offered the supervisors greater personal outcomes, as the process felt more real. The supervisors described that arranging oral interactive feedback made them feel valued and perceived it as personally rewarding. They realised that they could influence the students in a positive way and that their role was more important than first assumed. Many appreciated the opportunities to challenge the students, reflect together and receive insights into diverse viewpoints. They felt privileged when the students spoke frankly to them and appreciated that they could notice and address students’ feelings.

*‘Ok*,* now you’ve reflected upon this*,* but how did it make you feel? (…) And in that moment*,* we somehow engage in shadow boxing with that experience.’* (Supervisor 7).

The supervisors felt more vulnerable during oral conversations, as the setting also opened for instant feedback to them from the students, both constructive feedback and compliments. They valued the immediate response during oral interactive feedback and missed it when going back to written feedback. Also, the students’ reactions helped the supervisors to continuously self-evaluate, and they experienced this indirect feedback as both rewarding and meaningful, giving them a sense of mattering and mastery of the supervisor role. At the same time, they felt that there was a higher demand for qualifications and a risk of being criticised in a different way when sitting face-to-face with students.

*‘I think it perhaps required more of me personally to provide oral feedback*,* because you then risk that (…) also your critique can be criticised in a way.’* (Supervisor 2).

### Connecting being a supervisor and meeting patients as physicians

Several parallels between their experiences in the supervisor role and their future role as physicians were highlighted by the supervisors in the focus group discussions. This was related to leadership, communication and building relationships. The supervisors felt responsible for leading and concluding conversations within the time available, and they wanted to meet each student at their individual level. Encouraging students to provide feedback to themselves, mirrored a physician guiding a patient in self-reflection. Through oral interactive feedback, the supervisors practiced communication techniques, such as signposting, asking open ended questions, active listening and using silence. They underlined the importance both of using these communication techniques intentionally and recognising their role in creating a safe environment for students to share their thoughts, thereby influencing the key points of the feedback dialogue. Mastering these techniques was considered essential skills to becoming good physicians.

*‘And that I believe one will find use for later*,* simply daring to be a little bit quiet*,* just listening.’* (Supervisor 6).

The supervisor experience gave insights into many different understandings of the same patient. One of the supervisors noted that this enhanced the perception of how the same patient encounter can serve as origin to multiple interpretations. This realisation made the supervisor become humbler and more open to the possibility of being wrong in one’s own interpretation, acknowledging it is not the only interpretation, nor necessarily the correct one. The key takeaway from this experience was the importance of asking patients for clarification to ensure accurate understanding, rather than silently interpreting their statements.

*‘(…) maybe that I am not any better at interpreting things in one last way*,* which isn’t necessarily the correct answer. I guess it makes me more open to being wrong in the way I interpret them (…).’* (Supervisor 8).

Meeting the students through providing oral interactive feedback also gave the supervisors experiences in establishing professional relationships, something they highlighted as relevant to their future profession. They saw the feedback dialogues as unique opportunities to practice how to build a relationship from the ground, as such opportunities are limited before it is required of them as physicians. Through the oral interactive feedback, the supervisors risked finding themselves in uncomfortable situations, but this was portrayed positively, as they knew they could expect similar situations in their future profession. One of the supervisors referred to it as building a pedagogical alliance and compared this to a therapeutic alliance which will be important in their future role as physicians.

*‘Because they ought to trust you*,* because it is in a way*,* this is also often personal things we talk about*,* so it’s good to have*,* not a therapeutic alliance*,* but a pedagogical alliance to rely on.’* (Supervisor 7).

## Discussion

This study shows that through oral interactive feedback with near peers, medical students acting as supervisors in a reflective, person-centred context may develop their supervisor competency and evolve on a personal level including increasing their self-confidence. The supervisors experienced that oral interactive feedback is rewarding both professionally and personally, helping them draw lines to their future role as physicians.

These results correlate with previous research showing that acting as a peer supervisor supports development of professional skills crucial for future clinical careers, such as skills in leadership, communication, time-management, organising, feedback, reflection and critical thinking [3, 6, 9–11, 13, 20]. The supervisors in our study also saw that improvement in these skills was relevant for their future profession as physicians. According to Carless and Boud [9], the facilitation of feedback enhances learning strategies by fostering an appreciation for feedback processes, enabling individuals to make sound academic judgments, and allowing them to navigate the emotional dynamics in interpersonal communication.

Furthermore, literature suggests that student supervisors may be more qualified than experts in providing feedback to students due to their cognitive and social congruence with students. This congruence allows peers to better understand student challenges, fostering safe environments and genuine interest [3, 6, 7]. In our study some of the supervisors perceived that their supervisor tasks demanded higher qualifications than they felt they possessed. However, they found themselves exceeding their initial expectations, developing a sense of mattering and mastery of their role. This aligns with previous research indicating that students enjoy helping others when engaging in teaching [11, 13].

Mattering has numerous positive effects for both supervisors and institutions. Emotions, and also the experience of mattering, are closely related to learning, identity development and mental well-being [21–24], and may therefore positively impact the student supervisors. Facilitating peer feedback not only enables student supervisors to actively establish and develop their professional identity, but also helps them recognise their educational responsibilities as medical students today and as future physicians [20]. For institutions, positive effects arise for instance when supervisors choose to volunteer again due to positive experiences last time participating, reducing training time for new supervisors and increasing the competence of student supervisors. These institutional gains come in addition to the effects on the student supervisors mentioned above and the decrease in their risk of burnout [24].

One of the main challenges related to oral interactive feedback according to the supervisors in our study was logistics and time. This aligns with the results from a study from 2025, performed in Stockholm, where peer teachers and peer learners reported time and logistics as barriers for becoming, or continuing as a peer teacher [13]. In our study, the supervisors reported that both scheduling the meeting time, preparing for each meeting and having the feedback session itself were often time-consuming. They also worried whether the students would find the feedback worthwhile their time. For the students’ final reflective essay, the supervisors were asked to choose feedback method. One supervisor suggested allowing students to choose their preferred method, an idea supported by others. However, most of the supervisors opted for written feedback, because oral interactive feedback was too time-consuming, especially when closing in to exam periods. These decisions were made despite their own and existing literature’s conclusions about oral interactive feedback as the most effective method to stimulate students’ learning.

Optimal feedback should be continuous, arranged as soon as possible after a performed task, and enable learning outcomes to arise through dialogue [1, 8]. In our study, moving from oral interactive feedback back to written feedback was described by one of the supervisors like ‘closing the door’, suggesting that the lack of continuity in showing empathy and being person-centred was a negative experience for the supervisor. One could argue that maintaining continuity in the student-supervisor relationship may play a positive role also for the supervisors, not only for the students.

The supervisors in our study appreciated that oral interactive feedback made it easier to provide student-centred feedback and related this to physicians’ abilities to provide person-centred patient care. The supervisor role with oral interactive feedback offered practice in how to establish professional relationships and how to meet each student at their individual level. Literature emphasises this as important factors in feedback [1, 5, 8, 13]. Because the feedback dialogue in itself constantly provided feedback on how it functioned and where they were heading, the supervisors could calibrate to the student’s reactions by choosing words, tone and body language suitable for each specific context and person. It could be argued that practicing feedback skills in situations similar to physicians, results in solid foundations to provide better patient care in the future.

Furthermore, reflecting on their own feedback skills may help supervisors improve their feedback quality, which their future patients likely will gain from, in addition to positive effects for the supervisors themselves and their potential next students [7, 8]. In advance of our interviews, the supervisors had reflected on their experiences to some extent, but it was clear that the interviews functioned as additive reflective rooms, which they experienced as personally advantageous.

To some extent, many of our results mirror what has been found in other studies, but differ in relationship types and settings. Near-peer relationships may be different from peer-to-peer relationships, positioning our study to offer new perspectives, although we still assume that our findings are transferable to peer-to-peer settings as well. The settings of previous studies on peer teaching are typically courses where specific biomedical knowledge and performance are important [5–7, 10, 13]. In contrast, our study is set to a course with a different type of curriculum, concentrating on reflection, professional identity formation and empathy rather than specific measurable academic performances. Thus, our study provides new insights into development for student supervisors in this area of the medical curriculum. Nevertheless, our results may well be applicable to other feedback experiences in various settings, including other medical courses and other educational programs, not least where leadership, communication and safe relationships are important in their future professions.

### Limitations

As we have altered the way of feedback in the course and have trained the student supervisors before they experienced the change from written to oral interactive feedback, our study is not merely observational, but partly interventional. All authors are also deeply contextually involved, being former teachers and student supervisors in the course. On the positive side this gives us insight, making it easier to understand what informants are sharing and their context. On the other side it could possibly give other blind spots, including a risk of not exploring what we find to be obvious, and maybe not being able to describe all necessary aspects for the reader.

One may argue that our sample of 10 informants was sparse. We did, however, include 2/3 of the 15 eligible supervisors for the specific course and school year, in which we performed the study. We also had to take a pragmatic approach with consideration to practical aspects, leading to a varied number of informants in the focus group discussions (five, three, and two, respectively). We will argue that the information power of our sample was sufficient because of a narrow study aim, specific sampling, established theory supporting the analysis and strong dialogues [25].

### Future research

Future research should continue to investigate the learning outcomes and development for student supervisors and may dive deeper into near-peer oral interactive feedback or focus on other aspects of the supervisor role. Changing the settings to a different type of curriculum or change the group of interest, would also be of value.

### Implications

Implications of near-peer supervising can be described on three levels. Looking at existing literature, one could anticipate that introducing oral interactive feedback enhances the learning outcomes for the first level – the students taking the course, because the feedback can be more understandable, relevant and meaningful when provided by another student [1, 3].

On the second level, our study reveals that medical student supervisors can experience both professional and personal development and draw connections from their learning outcomes to their future role as physicians. This suggests that they develop abilities that help them become better physicians and may become more willing and capable to supervise others in the future. Also, student supervisors in other courses may experience similar development, which can be helpful in their future professions.

On the third level, institutions may gain from arranging near-peer feedback. Using students as supervisors can lighten the teaching burden when increasing student numbers become a challenge [1, 6, 7]. In addition, institutions can justify applying near-peer feedback as it underpins rewarding learning activities, not only for the attending students, but as our study has shown, also for the student supervisors.

This study has provided broader knowledge about students’ development from supervising near peers in a context of reflection, professional identity and empathy. By gaining a deeper understanding of how student supervisors learn from providing feedback, educators may design fulfilling near-peer feedback activities, fostering a reciprocal learning process from which both parts of the feedback process can enhance their learning outcomes.

## Conclusions

In conclusion, our study provides a deeper understanding of near-peer student supervisors’ potential development from providing oral interactive feedback. Not only do they acquire increased confidence, better supervisor competency and gains in professional and personal areas, but the development in their supervisor role has also helped them see parallels between their supervisor experiences and their future role as physicians. Nevertheless, this feedback approach can provide challenges, including logistical issues. We believe our findings are transferable to other contexts where reflection, development of professional identity, and empathy are central. Our findings align with previous literature from other settings and highlight the importance for institutions to arrange near-peer oral interactive feedback, including addressing potential challenges, as this educational strategy is associated with improved learning outcomes for both the attending student and the student supervisor.

## Supplementary Information

Supplementary Material 1: Interview guide.

## Data Availability

The datasets analysed during the current study are available in Norwegian from the corresponding author on reasonable request.
