# Supplementary material for: How near-peer supervisors experience their own development in the supervisor role when shifting from written to oral interactive feedback: a qualitative interview study
Source: BMC Med Educ. 2025 Aug 16;25:1168. doi: 10.1186/s12909-025-07798-0 (PMC12357462; doi:10.1186/s12909-025-07798-0)
Supplement: Supplementary file 1 — Supplementary Material 1: Interview guide. [file 12909_2025_7798_MOESM1_ESM.docx]

# Interview guide 1.0:

# PASKON-supervisors in oral interactive feedback

## Remember before every interview

- Request acceptance for recording the interview
- Inform about what will be done with the recordings (ANONYMITY)
- The guide is just an overview that reminds you of what important topics to cover in the conversation. Remember to cover all the topics, but don't be afraid to naturally follow up the development of the conversation
- Use techniques for active listening
  - Can you tell more about it?
  - "Mmm". "Yes".
  - Open and interested body language

- Let the interviewee answer the questions

## The questions must

- be simple
- invite to long answers
- not ask them to answer on behalf of a group - only on behalf of themselves
- preferably cover difficult topics
- have what or which formulations, rather than why or how

## Main question – ***not used directly in the interview***

*“How do student supervisors experience development in their own supervisor role when shifting from one-way written feedback to oral interactive feedback?”*

## ***Warm-up question - always start with this***

- Question going around the table - Is this your first year as PASKON supervisors or have you had this role earlier? (Identify voice/name in the beginning).
- How did you experience receiving feedback on your reflective essays when you had PASKON as first-year students?

## THEME 1:

- **What did you think when you heard about the change to oral interactive feedback?**
- How did you prepare for the oral interactive feedback sessions?
- What assessments did you make of the reflection note in advance and what did you want to achieve in the conversation?
- Can you tell us a bit about the oral interactive feedback sessions? (Everyone)?
- What did you think during the oral interactive feedback sessions? What did you feel?
- (Take the answer as a starting point) 🡪 What happened that made you experience it this way?

## THEME 2:

- What response did you receive from the students?
- What new insights have you earned from this experience?
  - Was there an immediate feeling?
  - And what do you think now that some time has passed since the conversations?

THEME 3:

- What was different about executing feedback this way, compared to written feedback?
- What did you learn? What gains did you obtain?
- What has this experience done to your supervisor competence?

## Final comments

- (For the last reflective essay in PASKON, you may choose the feedback method yourself. What do you think you want to choose – and why?).
- Is there anything else you would like to add?

# Interview guide 3.0:

# PASKON-supervisors in oral interactive feedback

## ***Warm-up question - always start with this***

- Question going around the table **- Is this your first year as PASKON supervisors or have you had this role earlier?** (*Identify voice/name in the beginning*).
- How did you experience giving written feedback on the reflective essays?

## THEME 1:

- **What did you think when you heard about the change to oral interactive feedback?**
- How did you prepare for the oral interactive feedback sessions?
- What assessments did you make of the reflective essays in advance and what did you want to achieve in the conversation?
- Can you tell us a bit about the oral interactive feedback sessions? (Everyone)?
- **What did you think during the oral interactive feedback sessions? What did you feel?**
- **(Take the answer as a starting point) -> What happened that made you experience it this way?**

THEME 2:

- **What was different about executing the feedback in this way, compared to written feedback?**
- **What did you learn? What gains did you obtain?**
- (How did you experience receiving feedback on your own feedback?)
- **What has this experience done to your supervisor competence?**
- **Have you experienced that giving oral interactive feedback has helped you develop in your own role as a supervisor? In what way?**
- **Is what you learn through your role as a supervisor relevant to your future job as a physician? In what way?**

## THEME 3:

- What response did you get from the students?
- What new insights have you earned from this experience?
  - Was there an immediate feeling?
  - And what do you think now that some time has passed since the conversations?

## Final comments

- For the last reflective essay in PASKON, you may choose the feedback method yourself. What do you think you want to choose (or what have you chosen) – and why?
- Is there anything else you would like to add?
